# Vitamin D: more does not mean better

**DOI:** 10.20945/2359-3997000000303

**Published:** 2020-10-08

**Affiliations:** 1 Universidade de São Paulo Faculdade de Medicina de Ribeirão Preto Departamento de Clínica Médica Ribeirão Preto SP Brasil Departamento de Clínica Médica, Faculdade de Medicina de Ribeirão Preto, Universidade de São Paulo, Ribeirão Preto, SP, Brasil

Science and technology have allowed human beings to live longer, more comfortably, and in relative safe life from natural weathering. In this scenario, non-communicable diseases linked to aging, sedentarism, and excess of energy consumption have become the major disorders to be contended with. Unfortunately, occasional movements against science have denied the benefit of historical conquests such as vaccination. Meanwhile, nutrition has a special appeal to the unscientifically minded; cyclically, a micronutrient emerges as a panacea able to guarantee health and protect against several chronic disorders. This thinking, similarly to the unfounded concern over vaccinations, tends to find basis in only observational studies with little other scientific backing. The last decade marked the turn of vitamin D. Previously considered a specialized molecule involved in the regulation of mineral metabolism, vitamin D is currently considered a pleiotropic hormone, as virtually all tissues and cells express vitamin D receptor ( 1 - 3 ). Subsequently, vitamin D deficiency started to be considered a cause for different disorders such as infection susceptibility, cancer, eclampsia, dental carie, cardiovascular diseases, autoimmune disorders, and diabetes mellitus type 1 and type 2 ( 1 ), and vitamin D, via supplementation, an easy solution. In all probability, the proposal ignores that, in the complex physiology of developed animals, there is no silver bullet able to cure disorders in all systems (e.g., immunity, cardiovascular, cell proliferation, endocrine and metabolic). It also does not take into consideration a simple rule that does not have exception: “more does not mean better”, i.e. not only deficiency, but also excess of every single essential element has detrimental effect, including those necessary for our survival. For instance, iron deficiency causes anemia, while excess of iron causes hemochromatosis; cortisol deficiency causes Addison disease, while excess of cortisol causes Cushing disease; even oxygen excess has adverse effects. Curiously, lack and excess of adipose tissue cause the same disorder, insulin resistance, while both vitamin D deficiency and excess have detrimental effects on bone.

In time of pandemic, researchers, physicians, and all chain of health employees are working hard to develop vaccine and drugs, as well as to assist patients harboring the SARS-CoV-2. Unexpectedly, they must also waste time alerting people to avoid false offers of therapy for prevention and treatment of COVID-19 that have no scientific basis and can have detrimental effects, including pharmacological dosage of vitamin D. In the present issue of the Archives of Endocrinology and Metabolism, Santos and cols. bring an appropriate view on the recommendation of vitamin D for those subjects showing vitamin D deficiency ( 4 ). More importantly, they call attention to the fact that boli of high and intermittent doses of vitamin D increase the risk of intoxication. Moreover, previous studies have shown that this regimen of vitamin D supplementation may increase falls and fractures.

Finally, I would like to give to the reader the consensual opinion from The International Osteoporosis Foundation (IOF), American Society for Bone and Mineral Research (ASBMR), American Association of Clinical Endocrinologists (AACE), Endocrine Society, European Calcified Tissue Society (ECTS), and National Osteoporosis Foundation (NOF) on usage of vitamin D in response to the current global pandemic, the COVID-19 ( 5 ). They alert that the safe exposure, avoiding sunburn, of skin directly to sunlight for only 15-30 minutes daily is the main source of vitamin D. They also consider that, in those countries where health authorities are recommending staying at home, limiting the possibility to acquire endogenous vitamin D, this could cause vitamin D deficiency. In this case, the six scientific and medical organizations recommend the intake of food with supplement of vitamin D and/or vitamin D nutritional supplements. The indicated dosage for adults 19 years and older varies from 400 to 1000 IUs, daily, according to sex and age, as indicated by the Institute of Medicine (IOM) ( 6 ). All these important societies, linked to bone and mineral metabolism, recognize the importance of adequate levels of vitamin D and calcium for bone mass development and maintenance. Also, they reason that experimental evidence suggests that vitamin D may influence immune response, which justifies further investigation into vitamin D supplementation for COVID-19. In unison, they worry about the repercussions of recent observational studies that reported an association of vitamin D deficiency with the SARS-CoV-2 infection, which is insufficient to establish a causal relationship. Indeed, vitamin D deficiency is a common trait of several diseases; in other words, vitamin D is a marker of health or illness ( 7 ). There is currently no randomized controlled study showing a beneficial effect of vitamin D supplementation on the prevention of COVID-19. In accordance to this awareness, there are consistent findings showing the inefficiency of higher doses of vitamin D on the prevention of infection. Rosendahl and cols. reported that 400 IU vitamin D3 seems adequate in maintaining vitamin D sufficiency in children younger than 2 years and that the increased dosage of 1200 IU neither ameliorated bone strength nor decreased infection ( 8 ). More recently, a clinical trial performed in Mongolia described that, compared to placebo, vitamin D supplementation among vitamin D – deficient schoolchildren in Mongolia (weekly oral dose of 14,000 IU of vitamin D3) did not reduce the risk of tuberculosis infection, tuberculosis disease, or acute respiratory infection ( 9 ).

In summary, safe exposition for few minutes (15-30 min) to sunshine naturally enables human beings to acquire the necessary amount of vitamin D, via auto sustainable synthesis of cholecalciferol. Alternatively, exogenous Vitamin D may safely bypass the thermal control of cholecalciferol synthesis within the skin when used in appropriate amounts. This target can securely be attained in most adults following the recommendation of IOM ( 6 ). The range of dosage suggested by Santos and cols. ( 4 ) is considered secure by the IOM ( 6 ).
